# Nanotherapeutic potential in glaucoma associated ocular cancers

**DOI:** 10.1007/s12672-025-04169-5

**Published:** 2026-01-28

**Authors:** Lidawani Lambuk, Muhammad Zulfiqah Sadikan, Mohd Aizuddin Mohd Lazaldin, Fatmawati Lambuk, Ramlah Kadir, Norzila Ismail, Rohimah Mohamud

**Affiliations:** 1 Optometry and Vision Science Programme, Center for Community Health Studies (ReaCH), Faculty of Health Sciences, Universiti Kebangsaan Malaysia, Jalan Raja Muda Abdul Aziz, 50300, Kuala Lumpur, Wilayah Persekutuan Kuala Lumpur Malaysia; 2 Department of Pharmacology, Faculty of Medicine, Manipal University College Malaysia, Jalan Batu Hampar, Bukit Baru, 75150 Melaka, Malaysia; 3 Department of Biosciences, Faculty of Science, Universiti Teknologi Malaysia, 81310 Johor Bahru, Johor Malaysia; 4 Department of Immunology, School of Medical Sciences, Health Campus, Universiti Sains Malaysia, Jalan Raja Perempuan Zainab 2, Kubang Kerian, 16150 , Kota Bharu, Kelantan Malaysia; 5 Department of Pharmacology, School of Medical Sciences, Universiti Sains Malaysia, 16150 , Kota Bharu, Kelantan Malaysia

**Keywords:** Nanotherapeutics, Glaucoma-associated ocular cancers, Ocular drug delivery, Theranostic, Targeted ocular therapy

## Abstract

Glaucoma-associated ocular cancers, including uveal melanoma and retinoblastoma, share overlapping pathological mechanisms that complicate diagnosis and treatment. Conventional imaging techniques, such as optical coherence tomography, often fail to distinguish glaucomatous neurodegeneration from tumour-induced damage, while standard chemotherapy and radiotherapy may exacerbate glaucoma progression. These diagnostic and therapeutic challenges highlight the urgent need for integrated management strategies. Recent advances in nanotechnology offer innovative solutions, with nanotherapeutics enabling targeted drug delivery, enhanced imaging contrast, and combined diagnostic–therapeutic (“theranostic”) capabilities. Various nanoparticle platforms have shown promise in improving ocular drug bioavailability, precision targeting, and real-time disease monitoring. This review synthesizes current progress in nanomedicine for glaucoma-associated cancers, emphasizing multifunctional nanoparticles that concurrently support tumour suppression and neuroprotection. Despite encouraging preclinical findings, significant challenges remain, including limited penetration across ocular barriers, uncertain long-term biocompatibility, and complex regulatory requirements. Continued interdisciplinary research integrating nanotechnology with gene editing and vector-based delivery systems may pave the way for personalized, vision-preserving therapies that redefine the treatment paradigm for glaucoma-associated ocular malignancies.

## Introduction

Glaucoma is a chronic, progressive eye disease and a leading cause of irreversible blindness worldwide, characterized by optic nerve damage often associated with elevated intraocular pressure (IOP) [1]. Known as the “silent thief of sight,” glaucoma progresses insidiously and often without symptoms, leading to irreversible vision loss before diagnosis. This asymptomatic onset poses major challenges for early detection and highlight its significance as a global public health concern [2]. Despite advancements in management, glaucoma remains incurable, highlighting the persistent need for innovative approaches [3].

This challenge is exacerbated when glaucoma overlaps with ocular cancers, such as uveal melanoma, retinoblastoma, and choroidal melanoma [4, 5]. Epidemiological and clinical evidences reveal the complex, bidirectional relationship where these conditions share overlapping pathological mechanisms, including inflammation, oxidative stress, and vascular dysregulation [4, 6–10]. This association is driven by tumour-induced mechanical effects, such as obstruction of aqueous outflow, and complications from standard cancer therapies. For instance, radiation can induce ischemia and neovascularization, leading to neovascular glaucoma (NVG) [11, 12]. Consequently, patients often experience a dual clinical burden, where overlapping manifestations such as vision loss and optic nerve damage complicate both diagnosis and management [13]. Moreover, therapeutic interventions for one condition such as radiotherapy for ocular melanoma or chemotherapy for retinoblastoma, may inadvertently induce or exacerbate secondary ocular complications, including glaucoma, cataract, non-proliferative retinopathy, and maculopathy [14]. Warda, O [15] further highlighted how visual function influences treatment decisions and emphasized the impact of therapy-related complications on long-term visual outcomes.

These diagnostic and therapeutic challenges are further exacerbated by the limitations of conventional methods. Standard imaging techniques often lack the sensitivity and specificity to distinguish glaucomatous neurodegeneration from cancer-related pathology [16, 17]. Similarly, drug delivery to posterior segment tumours remains highly inefficient, as topical and systemic routes fail to achieve therapeutic concentrations in the uveal and choroidal regions, and invasive injections carry significant risks [18–21].

Nanotechnology offers a paradigm-shifting solution. Nanoparticles (NPs) have emerged as promising carriers for ophthalmic applications, offering advantages such as mucoadhesive properties, enhanced drug stability, and targeted tissue penetration [22]. More importantly, multifunctional nanotherapeutics represent a transformative approach for managing complex diseases like glaucoma-associated cancers. These engineered systems integrate diagnostic and therapeutic capabilities into a single platform, enabling selective tumour targeting, precise delivery of therapeutic agents, and enhanced imaging contrast for real-time disease monitoring, all while minimizing adverse effects on glaucomatous conditions [23].

This narrative review addresses the gap created by the pathological convergence of these diseases. We synthesize the transformative potential of nanotherapeutics, with a focus on pioneering platforms such as gold nanoparticles (AuNPs) for photothermal ablation and imaging, nanoparticle-based gene therapy for precise oncogene silencing, and virus-like particles (VLPs) for highly specific tumour targeting. The core novelty lies in designing single nanoscale systems capable of concurrently delivering chemotherapeutic agents to tumours and neuroprotective drugs targeting optic nerve. However, significant challenges impede its clinical translation. Hence, his review provides a critical evaluation of these theranostic strategies, not only highlighting their potential for a paradigm shift towards personalized, vision-preserving therapies but also addressing the hurdles of ocular barrier penetration, long-term biocompatibility, immune clearance, and scalable manufacturing. This review addresses these challenges to establish a practical framework for the integrated management of glaucoma-associated ocular cancers.

### Imaging and diagnosis

Ocular cancers, including those associated with secondary glaucoma, are often difficult to detect due to subtle and nonspecific symptoms, leading to diagnostic delays [24]. Although many tumours are non-metastatic at initial diagnosis, approximately 50% of patients eventually develop metastases, with the liver being affected in up to 90% of cases [25]. Glaucoma can develop when metastases invade the anterior chamber angle, obstructing aqueous outflow [26]. This is particularly common in tumours such as uveal melanoma or metastases from lung or breast cancer [27]. Once metastasis occurs, prognosis is poor, with a median survival time of less than one year, even with treatment [25]. Thus, early diagnosis of glaucoma-associated cancers is critical for improving patient outcomes.

Conventional imaging techniques play a vital role in detecting ocular cancers but are limited by insufficient resolution, low sensitivity, and the inability to visualize functional changes associated with malignancy. Table 1 summarizes these limitations.

Although conventional imaging techniques are essential for ophthalmic diagnosis, each modality has limitations, particularly in differentiating glaucomatous changes from malignancy. While other advanced modalities such as high-frequency ultrasound and emerging techniques like photoacoustic imaging, offer advantages in penetration depth and functional contrast, their routine clinical use for this specific diagnostic dilemma is not yet well established. The limitations of the most commonly used clinical techniques, as outlined in Table 1, therefore create a significant opportunity for nanotechnology-enhanced imaging. Nanotherapeutics, which involve NPs engineered for targeted drug and imaging agent delivery, offer significant advantages over traditional methods [28].

NPs composed of lipids, polymers, or metals improve drug solubility, prolong circulation time, and enhance specificity for target tissues [29, 30]. In the context of glaucoma-associated cancers, NP-based imaging agents facilitate early detection by selectively accumulating in affected ocular tissues. These imaging agents, including fluorescent dyes, radioactive isotopes, and MRI contrast agents, enhance visualization of cancer cells, enabling precise staging and diagnosis [31]. For example, fluorescent dyes conjugated to NPs can bind cancer-specific receptors, providing high-specificity imaging. Additionally, multimodal imaging approaches that combine fluorescent dyes and MRI contrast agents improve detection accuracy by offering both optical and anatomical insights [32].

Several factors must be considered when designing NP-based imaging systems. Lipid-based nanoparticles, such as liposomes and solid lipid NPs, are particularly advantageous due to their ability to encapsulate both hydrophilic and hydrophobic imaging agents [33–35]. Other crucial design parameters include optimizing NP size (preferably < 200 nm for enhanced cellular uptake and ocular tissue distribution) and surface charge (positively charged NPs improve cellular uptake through electrostatic interactions) [36–39]. Tissue penetration and targeted distribution of imaging agents are also essential for visualizing deeply located ocular cancer cells [40, 41].

Beyond cancer diagnosis, nanotechnology has the potential to improve glaucoma management. For example, nanosensors embedded in contact lenses or implanted within the eye can continuously monitor IOP, providing real-time data on disease progression [42]. These technologies contrast with traditional tonometry, which is often invasive and limited to clinical settings [43]. Some nanotechnologies integrate IOP monitoring with therapeutic functions, enabling precision drug delivery based on real-time pressure changes. These innovations, summarized in Table 2, represent a significant advancement in personalized glaucoma treatment.

NP-based imaging and monitoring technologies provide a powerful platform for the early detection and management of glaucoma-associated cancers. By carefully selecting nanomaterials, imaging agents, and multimodal approaches, these technologies can enhance diagnostic accuracy and improve patient outcomes. The integration of NP-based imaging with therapeutic strategies represents a significant step toward precision medicine in ocular oncology.

**Table 1:** Conventional diagnostic techniques for Glaucoma-associated cancers

Imaging method	Function	Limitation
OCT	Provides high-resolution, cross-sectional images of ocular tissues, primarily identifying changes in retinal nerve fiber layer (RNFL) thickness.	Resolution: 1–15 μm. Penetration: 1–2 mm. Sensitivity High for structural changes, but low for functional or metabolic assessment. Limited in detecting small tumours, as it mainly visualizes structural rather than functional changes [44].
Fundus Photography	Captures detailed images of the posterior segment of the eye to identify abnormalities, including tumours.	Resolution: ~10–20 μm. Penetration: Superficial layers only. Sensitivity: Moderate for large, pigmented tumours, low for small, amelanotic, or subretinal tumours. Lacks in depth perception and struggles to visualize small tumours[45].
Ultrasound Biomicroscopy (UBM)	Utilizes high-frequency ultrasound waves to provide detailed images of ocular structures.	Resolution: 20–50 μm. Penetration: 4–5 mm. Sensitivity: High for anterior segment tumours. Low for posterior segment tumours due to signal attenuation [46].
Fluorescein Angiography (FA)	Uses intravenous fluorescent dye to highlight blood vessels in the eye, aiding in detecting neovascularization.	Resolution: Dependent on underlying fundus camera. Penetration: Primarily retinal vasculature. Sensitivity: High for vascular leakage and neovascularization. Ineffective in visualizing non-vascular or poorly perfused tumours [47].
Indocyanine Green Angiography (ICG)	Uses indocyanine green dye to image choroidal blood vessels that can be helpful in identifying tumours with choroidal involvement.	Resolution: Dependent on underlying fundus camera. Penetration: Deeper choroidal vasculature. Sensitivity: High for choroidal pathologies. Limited in detecting tumours affecting other eye structures and in differentiating benign from malignant lesions. [48, 49].
Magnetic Resonance Imaging (MRI)	Provides detailed anatomical and structural information.	Resolution: 100–500 μm. Penetration: Full orbit and brain. Sensitivity: Low resolution for small ocular structures. Requires contrast agents for better visualization of tumour vascularity and extent [50].
Computed Tomography (CT)	Useful for evaluating orbital tumours and bony structures.	Resolution: 200–500 μm. Penetration: Full orbit and bone. Sensitivity: Limited sensitivity for small intraocular tumours and potential radiation exposure. Best for detecting calcification like in retinoblastoma and bone erosion. [51].
Slit-lamp Biomicroscopy	Examines ocular structures using a slit-like beam of light; it is a valuable technique for basic evaluation.	Resolution: ~10–20 μm. Penetration: Anterior segment only. Sensitivity: Low for internal and posterior segment details. Insufficient resolution for detecting small or deep-seated tumours [52].

**Table 2:** Revolution in glaucoma treatment by combining IOP monitoring

Nanotechnology	Function
Implantable pressure sensors	Tiny biosensors made of nanoparticles that wirelessly transmit IOP data to an external device [53]
Microfluidic Contact Lenses	Lenses embedded with microfluidic channels to measure IOP changes based on fluid flow in the eye [54]
Nanoparticle-based sensors	Nanoparticles injected into the eye that change colour or fluorescence in response to IOP fluctuations, allowing non-invasive monitoring [42]

### Therapy

Treating cancers in immune-privileged organs like the eye is challenging due to anatomical constraints and proximity to critical structures, complicating surgery and drug delivery [55]. The fragility of ocular tissues limits aggressive treatments, particularly for glaucoma-associated cancers. Additionally, ocular barriers including the corneal epithelium, blood-aqueous barrier, and blood-retina barrier, impede drug penetration, reducing the efficacy of topical and systemic therapies. The cornea, a five-layered transparent structure, serves as a primary drug barrier [56]. Tight junctions in the epithelium restrict hydrophilic molecules, while the stroma’s charged collagen fibrils limit hydrophobic compound diffusion [57]. Beyond the cornea, the blood-aqueous barrier (non-pigmented ciliary epithelium) regulates drug entry into the anterior chamber via active and passive transport. The blood-retina barrier (inner and outer layers) selectively filters systemic drugs, preventing access to the retina and vitreous [58, 59]. Additionally, pre-corneal clearance mechanisms, tear turnover, blinking, nasolacrimal drainage, and tear film restoration, rapidly eliminate topically applied drugs, leaving less than 5% to reach ocular targets [60].

### Current treatment for ocular cancers

#### Surgical therapy

Surgical intervention is a primary treatment option for ocular cancers, particularly in cases where tumours are localized or unresponsive to other therapies. The choice of surgery depends on tumour size, location, and progression. Enucleation, which involves the complete removal of the eye, is often necessary for advanced retinoblastoma or uveal melanoma that cannot be managed with other treatments [61]. More extensive cases require exenteration, where the entire orbital content, including the muscles, fat, and optic nerve, is removed, a procedure used in aggressive and invasive orbital malignancies [62]. For smaller tumours, iridectomy and iridocyclectomy involve the partial removal of iris tissue to preserve vision while eliminating cancerous growths [63]. Plaque brachytherapy, a specialized approach combining surgery and radiation, involves the placement of a radioactive plaque directly on the tumour to deliver localized radiation while minimizing damage to healthy tissues [64]. Vitrectomy, a technique used for intraocular tumours, removes part or all of the vitreous humour, sometimes combined with intravitreal chemotherapy for better treatment outcomes [65]. Additionally, emerging treatments like cryosurgery, which uses extreme cold to freeze and destroy cancer cells. While surgical approaches can successfully control tumours, they may lead to vision loss or cosmetic challenges, necessitating prosthetic rehabilitation or reconstructive surgery in some patients [66].

#### Chemotherapy

Chemotherapy is commonly used in treating retinoblastoma and certain ocular adnexal tumours. Systemic chemotherapy, using agents such as carboplatin, vincristine, and etoposide, is frequently employed for retinoblastoma, though it has limited efficacy in treating uveal melanoma [67]. More localized methods, such as intravitreal chemotherapy, allow for the direct injection of melphalan or topotecan into the vitreous cavity, achieving high drug concentrations within the eye while reducing systemic side effects [68]. Additionally, periocular chemotherapy, including sub-Tenon carboplatin injections, enhances drug penetration into intraocular tissues [69].

#### Radiotherapy

Radiotherapy is an essential treatment for uveal melanoma, choroidal melanoma and retinoblastoma, particularly when tumours are not surgically accessible. Laser ablation offers a less invasive alternative for localized tumours by using high-intensity laser beams to destroy cancerous tissue. However, the procedure risks damaging adjacent healthy ocular structures and is ineffective for deeper tumours, such as those in the choroid or retina [70].

Localized radiotherapy, plaque brachytherapy is a widely used method, employing iodine-125 or ruthenium-106 plaques to deliver targeted radiation directly to the tumour site, sparing adjacent healthy tissues [71]. External beam radiotherapy (EBRT) is often used for bilateral retinoblastoma, though it carries risks of radiation retinopathy and cataract formation [71]. More advanced techniques such as proton beam therapy (PBT) allow for high-precision radiation delivery, minimizing collateral damage to surrounding structures [72]. Recent advances in minimally invasive surgical techniques have improved tumour removal precision while reducing ocular trauma. Techniques such as precision-guided laser surgery and robotic-assisted surgery allow for millimeter-level accuracy, minimizing damage to healthy tissue [73]. However, radiation exposure can damage surrounding tissues, leading to complications such as cataracts, radiation retinopathy, or optic neuropathy [71].

#### Targeted therapy and immunotherapy

Molecularly targeted therapy has gained interest in managing ocular cancers, particularly uveal melanoma, which is often resistant to conventional treatments. Inhibitors targeting GNAQ/GNA11 mutations, commonly found in uveal melanoma, are under investigation [74]. Tyrosine kinase inhibitors (TKIs), such as sunitinib and imatinib, have shown potential in treating ocular adnexal malignancies, though their ocular penetration remains a challenge [75].

Immunotherapy, particularly immune checkpoint inhibitors (ICIs) like pembrolizumab and ipilimumab, has demonstrated promising outcomes in metastatic uveal melanoma [76]. However, ocular tumours exhibit immune privilege, limiting the effectiveness of systemic immunotherapy. Intravitreal administration of immune-modulating agents is an emerging approach to enhance local immune responses while reducing systemic toxicity.

### Challenges in conventional ocular drug delivery

Despite advancements in treatment, ocular drug delivery remains a significant challenge, primarily due to the eye’s intricate anatomical and physiological barriers. Topical administration, the most common method, is severely limited by rapid pre-corneal clearance, while systemic administration suffers from poor ocular bioavailability, with typically less than 2% of the administered dose reaching the retina [77]. While more direct, invasive procedures like intravitreal injections carry substantial risks, including endophthalmitis, retinal detachment, and cataract formation [78]. Periocular injections (e.g., sub-Tenon, retrobulbar) offer a less invasive alternative with better penetration than topical routes, but are still challenged by limited drug retention and distribution to the target site [79]. These limitations highlight the critical need for more sophisticated drug delivery strategies.

### Nanotechnology as a strategic solution

To overcome these hurdles, nanotechnology-based platforms have emerged as a paradigm-shifting approach. Engineered nanocarriers are designed to address the specific shortcomings of conventional methods through several key mechanisms [23]. Their nanoscale size and tunable surface chemistry, including the use of mucoadhesive polymers, enhance corneal residence time and facilitate penetration across the blood-aqueous and BRB [22, 80]. A pivotal advantage is their capacity to actively target by modifying surface with ligands such as peptides, antibodies, or aptamers, allowing nanoparticles to attach to receptors that are overexpressed on tumour cells.

This enables site-specific delivery, maximizing therapeutic impact on cancerous tissue while rigorously minimizing off-target effects on critical healthy structures like the optic nerve and retinal ganglion cells, which are particularly vulnerable in the context of co-existing glaucoma [81]. For a comprehensive recent review on active targeting strategies, readers are directed to Trivedi, N [82]. This targeting capability represents a fundamental shift from the untargeted cytotoxicity of standard chemotherapeutics towards a precision medicine approach.

Furthermore, nanoparticles act as sustained-release reservoirs, providing controlled drug delivery over extended periods. This can drastically reduce the frequency of invasive injections and improve patient compliance [83]. Looking beyond conventional chemotherapeutics, these versatile platforms are also being engineered for gene therapy and CRISPR-based approaches, enabling direct targeting of oncogenic mutations in ocular tumours [84].

This strategic potential is further amplified when combined with other advanced modalities, such as biodegradable implants for long-term release and gene therapy for precise molecular intervention [85, 86]. However, the following section will provide a detailed examination of the most promising multifunctional nanotherapeutic platforms, including gold nanoparticles, gene therapy vectors, and virus-like particles, for the integrated management of glaucoma-associated ocular cancers.

### Multifunctional nanotherapeutics

Nanotechnology has transformed ocular drug delivery, offering innovative solutions for conditions like glaucoma and ocular cancers, which are highly associated due to shared pathophysiological mechanisms and therapeutic challenges, as noted by Kagkelaris, K [87]. Since the 1980s, nanoparticle-based systems have harnessed advances in materials science, physics, chemistry, and biology to enhance drug solubility, stability, and targeted delivery, overcoming the limitations of conventional therapies. These systems include organic NPs such as liposomes, dendrimers, and nanoemulsions, as well as inorganic NPs like metallic, carbon-based, silica, and quantum dots [88–90]. Organic NPs primarily serve as drug carriers, while inorganic NPs offer imaging capabilities [88]. For example, mesoporous silica NPs can encapsulate high drug loads, enabling sustained release [91]. NPs, whether organic, inorganic, or hybrid [92], facilitate targeted delivery through solubilization, encapsulation, or chemical bonding, making them ideal for addressing the interconnected challenges of glaucoma and ocular cancers [93]. Additionally, biologically derived NPs, such as exosomes and microvesicles, show promise in gene delivery, immunomodulation, and tissue regeneration, which are critical for managing both conditions.

The high association between glaucoma and ocular cancer stems from shared risk factors, such as elevated IOP and chronic inflammation, as well as overlapping molecular pathways that contribute to disease progression. For instance, glaucoma features elevated IOP can create a microenvironment that promotes tumourigenesis, increasing the risk of ocular cancers like uveal melanoma [94]. Conversely, ocular cancers can induce secondary glaucoma by obstructing aqueous outflow or causing neovascularization, further complicating disease management [13]. This bidirectional relationship necessitates integrated treatment strategies, where nanotechnology plays an essential role. Multifunctional NPs are particularly effective, as they can simultaneously target IOP regulation in glaucoma and tumour suppression in ocular cancer. In glaucoma, NPs deliver drugs to critical structures like the trabecular meshwork and optic nerve head, improving bioavailability and reducing systemic side effects [22]. In ocular cancer, these NPs minimize off-target toxicity and preserve healthy tissues [81]. The shared need for sustained drug delivery has led to the adaptation of controlled-release formulations, originally developed for cancer, into glaucoma management, ensuring prolonged therapeutic effects and better patient adherence [95].

The overlap between glaucoma and ocular cancer treatments is evident in gene therapy-based nanotherapeutics. DNA tetrahedral NPs and DNA-based agents target glaucoma-related pathways, such as IOP regulation, while also suppressing tumour-specific genes in ocular cancer [96]. These shared NP platforms enhance the eye’s immune environment, reducing immune toxicity in both conditions [97]. Additionally, dual-functional NPs combining therapeutic payloads with imaging contrast agents enable real-time disease monitoring, essential for managing glaucoma’s optic nerve damage and ocular cancer’s tumour growth [94]. Protecting the optic nerve, crucial in glaucoma, is equally vital in ocular cancer to prevent vision loss, highlighting the need for integrated approaches [98].

Supporting this, Saurabh, R [99] review hybrid nanoparticles (HNPs) for cancer theranostics, highlighting their potential for targeted drug delivery, enhanced bioavailability, and minimized systemic toxicity in glaucoma. HNPs also improve cancer treatment by reducing off-target effects and ensuring drug specificity to cancerous tissues. Furthermore, controlled-release formulations for sustained drug delivery in both conditions reinforce the need for prolonged therapeutic effects and improved patient adherence. This comprehensive review emphasizes HNPs’ role in advancing personalized medicine for glaucoma and ocular cancer.

Despite these advancements, challenges persist due to the intricate relationship between glaucoma and ocular cancer. Their overlapping pathophysiology increases the risk of adverse effects, such as secondary glaucoma in cancer patients or an elevated cancer risk in glaucoma patients with chronic inflammation. Real-time disease monitoring and the safety of repeated NP administration remain concerns, with current research showing variable progress in ensuring long-term efficacy. However, NPs offer promising solutions by minimizing toxicity, enhancing drug delivery, and enabling precision medicine, making them integral to future integrated treatment strategies.

**Fig. 1:**
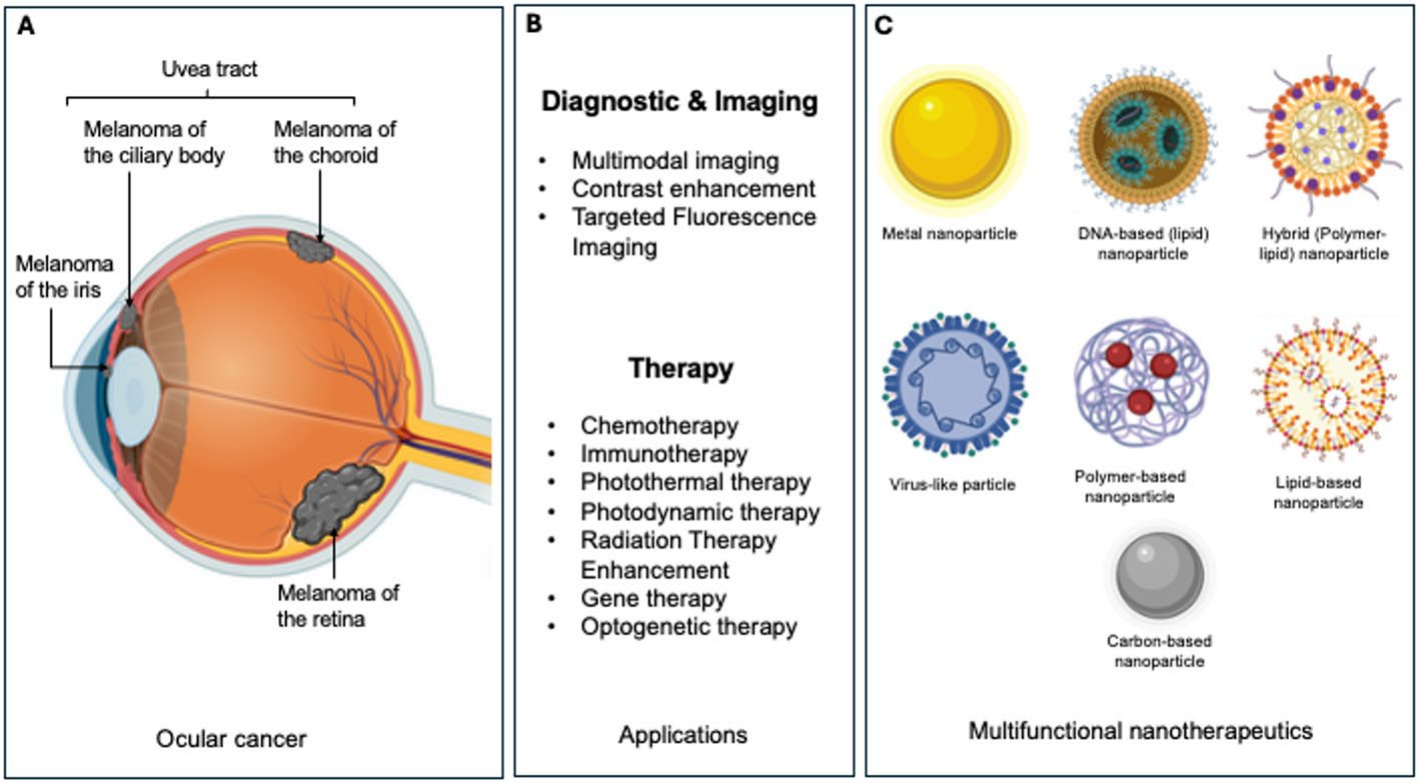
Schematic overview of multifunctional nanotherapeutics for the diagnosis and treatment of ocular cancers.** A** Overview of the eye anatomy affected by various ocular cancers, such as uveal melanoma located in the iris, ciliary body, and choroid, as well as retinoblastoma of the retina;** B** The landscape of nanotherapeutic platforms and their applications.** C** A diverse array of engineered nanoparticles, including lipid-based, polymer-based, metallic, carbon-based, viral-like, DNA-based, and hybrid nanoparticles, serve as versatile carriers. These platforms can be functionally tailored for a wide range of applications, from enhanced diagnostic and imaging to targeted therapy. The convergence of these functionalities into single systems enables the development of multifunctional nanotherapeutics, which can simultaneously diagnose, deliver treatment, and monitor response, representing a paradigm shift in managing glaucoma-associated ocular cancers.

In sum, the strong association between glaucoma and ocular cancer emphasize the need for unified therapeutic approaches, where nanotechnology presents transformative potential. Multifunctional NPs address shared challenges by enabling targeted drug delivery, sustained release, and real-time monitoring while preserving critical ocular structures like the optic nerve. Figure 1 provides a comprehensive overview of the current landscape and Table 3 summarizes recent studies investigating nanoparticles for multimodal imaging and therapy in ocular cancers.

**Table 3:** Recent studies done on nanoparticles for multimodal and therapy alone for ocular cancers

Nanoparticle Type	Application type	Imaging Modality	Stage	Target Condition	Outcomes	Limitations	References
Gold NPs (Brachytherapy)	Radiation therapy	CT	Preclinical (in vitro); Preclinical (in vivo)	Retinoblastoma	Enhances tumour radiation efficacy; spares healthy tissue	Retinal complications, cytotoxicity risks; off-target delivery	[100]
Magnetic Hollow Au Nanocages	PTT and immunotherapy	PA/US/MR	Preclinical (in vitro); Preclinical (in vivo)	Retinoblastoma	Improves PA/US/MR imaging; combines LIFU therapy and immunotherapy	Variable tumour response; complex synthesis	[101]
Folate/Magnetic Nanoliposomes	Chemotherapy	MRI / Fluorescence	Preclinical (in vitro); Preclinical (in vivo)	Retinoblastoma	95% cell uptake; near-complete tumour regression in mice	Requires human trials; stability concerns	[102]
Folate-Targeted Liposomes	PDT	Fluorescence	Preclinical (in vitro); Preclinical(in vivo)	Retinoblastoma	Laser-activated tumour destruction; no systemic toxicity	Targeting varies with tumour traits	[103]
MXene Quantum Dots	PTT	Photoacoustic	Preclinical(in vitro)	Uveal Melanoma	High tumoricidal activity; biocompatible	No human data; unvalidated imaging	[104]
DNA EG@Dual/hMNs NPs	Gene therapy	Bimodal	Preclinical(in vitro)	Retinoblastoma	Bimodal imaging; dual-gene therapy	Delivery/stability challenges; needs in vivo validation	[105]
CuFeSSe NPs	PTT	Photoacoustic	Preclinical(in vivo)	Uveal Melanoma	Hyperthermia/ROS-driven cuproptosis/pyroptosis	Toxicity risks; targeting precision needed	[106]
Cisplatin Nanoliposomes	Chemotherapy	N/A	Preclinical (in vitro); Preclinical(in vivo)	Retinoblastoma	High apoptosis; superior tumour reduction	No long-term toxicity/recurrence data	[107]
Abraxane (Albumin-Paclitaxel)	Chemotherapy	N/A	Clinical(Phase 2; NCT00738361)	Metastatic Ocular Melanoma	Improved solubility/targeting	Neuropathy/neutropenia; IV required	[108]
Belzupacap Sarotalocan (AU-011) VLP	PDT	N/A	Clinical(Phase 2; NCT04417530)	Ocular Melanoma	High tumour control; vision preservation	Limited tumour criteria; moderate adverse effects	[109]
Camouflage NPs (Optogenetic)	Optogenetic therapy	N/A	Preclinical (in vitro)	Retinoblastoma	Precise optogenetic activation	Retinal cell impact; uptake efficiency	[110]
miRNA-Based Gene Therapy Nanodrug	Gene therapy	N/A	Preclinical(in vitro)	Ocular Melanoma	Suppresses melanoma growth, induces apoptosis, enhances immune response	Short tumour retention, frequent injections required, uncertain human translation	[111]
PLGA NPs **(**Melphalan)	Chemotherapy	N/A	Preclinical (in vitro)	Retinoblastoma	95% drug loading; sustained release; ultrasound-enhanced uptake	Requires in vivo testing; toxicity at high doses	[112]
Lipid NPs (black phosphorus quantum dots /melphalan)	Chemo-PTT	N/A	Preclinical(in vitro)	Retinoblastoma	good stability and encapsulation efficiency; inhibits tumour proliferation	Requires human trials; stability/scaling challenges	[113]
Gold NPs (Rosiglitazone)	Chemotherapy	N/A	Preclinical(in vitro)	Retinoblastoma	Reduces proliferation and induces apoptosis	Lacks in vivo validation, off-target effects, large-scale production challenges	[114]
Hyaluronic Acid NPs (Verteporfin)	PDT and immunotherapy	N/A	Preclinical (in vitro); Preclinical (in vivo)	Uveal Melanoma	High tumour accumulation; ROS-driven immunity	Requires laser; lacks metastatic data	[115]
Galactose-Conjugated Polymeric NPs (Etoposide)	Chemotherapy	N/A	Preclinical(in vitro)	Retinoblastoma	Sustained drug release, improved cytotoxicity, localized effect	No in vivo validation, stability, scalability issues	[116]
Chitosan NPs (Topotecan)	Chemotherapy	N/A	Preclinical (in vitro); Preclinical(in vivo)	Retinoblastoma	High encapsulation, enhanced cytotoxicity, significant tumour reduction	Ocular toxicity potential, scalability challenges	[117]
NLCs (haloperidol metabolite II valproate ester- MRJF22)	Chemotherapy	N/A	Preclinical (in vitro)	Uveal Melanoma	Potential therapeutic strategy via ophthalmic delivery of haloperidol metabolite	Requires efficacy/safety validation; scalability challenges	[118]

*PTT*: Photothermal Therapy,* PDT*: Photodynamic Therapy,* ROS*: Reactive Oxygen Species,* MRI*: Magnetic Resonance Imaging,* CT*: Computed Tomography,* PA*: Photoacoustic Imaging,* US*: Ultrasound,* AuNPs*: Gold Nanoparticles,* VLP*: Virus-Like Particle,* PLGA*: Poly(Lactic-co-Glycolic Acid),* NLCs*: Nanostructured Lipid Carriers,* LIFU*: Low-Intensity Focused Ultrasound,* N/A*: Not Applicable

### Biocompatible nanomaterials

The eye’s extreme sensitivity and limited regenerative capacity make superior biocompatibility a fundamental prerequisite, not just a regulatory hurdle for any clinically viable nanotherapeutic. Apart from the elevated IOP, introducing foreign materials risks inflammation, clouding of optical media or irreversible nerve damage, directly undermining the goal of vision preservation in glaucoma-associated cancers. Among the array of materials investigated, several frontrunners have emerged, distinguished by their proven safety profiles and functional versatility for ocular oncology applications.

Lipid-based nanocarriers, including liposomes, solid lipid nanoparticles (SLNs), and nanostructured lipid carriers (NLCs), currently represent the frontrunner for ocular drug delivery. Their compositional similarity to biological membranes underlies exceptional tolerability, while their amphiphilic structure enables encapsulation of both hydrophilic and hydrophobic drugs. Advances in lipid engineering have produced cationic and surface-functionalized liposomes capable of corneal adhesion, controlled release, and tumour-specific delivery, as demonstrated by folate-targeted liposomes for photodynamic therapy in retinoblastoma [103]. The soft, flexible nature of these carriers minimizes mechanical trauma to delicate retinal tissues. Despite these advantages, lipid-based nanocarriers remain limited in theranostic applications compared to inorganic systems. Their inherently soft and optically inert structure restricts their use in diagnostic imaging, often leading to signal instability or rapid leakage of imaging agents. Furthermore, their mechanical fragility and biodegradability can compromise stability during laser exposure and lead to short intraocular residence times.

In contrast, AuNPs are the leading inorganic platform for theranostic applications that integrate ocular imaging and treatment. They possess intrinsic optical and photothermal properties, offering superior imaging contrast, energy conversion efficiency, and structural stability under therapeutic irradiation. Although non-biodegradable, their surfaces can be functionalized with polyethylene glycol (PEG) or tumour-targeting ligands to improve ocular biocompatibility and limit non-specific uptake. As summarized in Table 3, AuNPs have been successfully deployed as radiosensitizers and photothermal ablation cores. Recent studies have also explored hybrid lipid–AuNP systems, merging the biocompatibility of lipids with the imaging potency of gold to create multifunctional theranostic nanoplatforms [87].

Among synthetic polymers, Poly(Lactic-co-Glycolic Acid) (PLGA) remains a preeminent candidate owing by the US FDA and European Medicine Agency (EMA)-approved status in various drug delivery systems in humans and predictable. It enables controlled, sustained drug release, which is critical for maintaining therapeutic intraocular levels while reducing invasive injection frequency (Weng et al., 2017; Gote et al., 2019). For instance, PLGA NPs encapsulating melphalan for retinoblastoma achieved high drug loading and prolonged release [112]. Nevertheless, acidic degradation byproducts can occasionally induce mild vitreous inflammation, necessitating pH-stabilizing co-formulations.

Meanwhile, Hyaluronic Acid (HA), a naturally occurring polysaccharide and a component of the vitreous humour, offers unmatched innate biocompatibility and active targeting potential via the CD44 receptor, commonly overexpressed in uveal melanoma. HA nanoparticles loaded with verteporfin demonstrated enhanced tumour accumulation and effective photodynamic tumour suppression [115]. Its physiological compatibility minimizes interference with optical function, positioning it as a highly safe and targeted vehicle.

Taken together, the frontrunners in ocular nanotherapy represent a strategic convergence of safety, versatility, and functionality. Lipid-based nanocarriers celebrated for their proven biocompatibility and clinical translatability, stand as the primary workhorse for targeted drug delivery. AuNPs with their unique physicochemical properties, are the undisputed leaders for pioneering integrated imaging and theranostic applications. Meanwhile, polymeric and hybrid systems expertly bridge these domains, offering controlled degradation and molecular targeting for sustained release. The field is increasingly adopting a “horses for courses” approach, where the ideal platform is selected based on the specific clinical challenge. For the dual pathology of glaucoma-associated cancers, the ideal nanocarrier must therefore harmonize this therapeutic potency with ocular inertness to achieve precise tumour eradication while preserving vital visual function. The following section evaluates how these platforms are specifically applied to target the complex pathophysiology of glaucoma-associated ocular cancers for enhanced diagnosis and therapy.

### Stimuli-responsive nanoparticles

Having established the leading biocompatible nanomaterials for ocular therapy, the next critical advancement lies in bestowing them with intelligence. The frontier of nanotherapeutics for complex dual pathologies like glaucoma-associated cancers is defined by smart ‘stimuli-responsive’ nanoparticles. By incorporating responsive elements into the structure of lipid NPs, AuNPs, or polymer-based systems like PLGA, these platforms can be transformed from simple carriers into targeted, on-command drug delivery systems. This intelligent responsiveness ensures precise spatiotemporal control, which is paramount for managing two concurrent conditions where localized and timed drug delivery is crucial [119]. For example, smart mesoporous silica nanoparticles (MSNs) and related nanocarriers can release their loaded medications in response to a variety of stimuli including changes in pH, redox reactions, enzyme activity, temperature, magnetic fields, or light exposure [120]. This capability for precise spatiotemporal control is particularly valuable in managing complex dual pathologies, such as glaucoma-associated cancers, where localized and timed drug delivery is crucial.

Internally responsive systems can exploit pathological features such as the acidic pH of diseased ocular tissues or the overexpression of enzymes like matrix metalloproteinases (MMP-9), as demonstrated in enzyme-responsive nanocarriers that release antimicrobials in bacterial keratitis [121] or in pH-sensitive vehicles that effectively deliver siRNA to inhibit corneal neovascularization [122]. Moreover, temperature-responsive nanogels have recently been developed as in situ ocular delivery systems, exhibiting a sol–gel transition at ocular-compatible temperatures to prolong drug residence time. For instance, a thermosensitive ketoconazole nanoparticle-loaded gel demonstrated enhanced solubility, sustained release, and strong antifungal activity in treating fungal keratitis [123].

In contrast, externally applied stimuli such as light or ultrasound, can be harnessed to precisely activate nanotherapeutics at targeted sites and times. Light-responsive polymeric nanoparticles have recently been investigated for retinal drug delivery, enabling controlled and on-demand release of therapeutic agents upon illumination while reducing off-target effects and phototoxicity [124]. Similarly, ultrasound-responsive nanobubbles have demonstrated enhanced intravitreal drug migration in ex vivo models, offering a non-invasive strategy to improve the distribution and penetration of therapeutic agents within posterior ocular tissues [125]. This intelligent responsiveness ensures maximal therapeutic impact on diseased regions while rigorously preserving the function of critical ocular structures, representing a promising step toward precise and vision-preserving nanotherapy.

#### Potential of gold nanoparticles

The ocular nanomedicine arsenal comprises diverse nanocarriers such as biodegradable polymeric nanoparticles for sustained drug release and solid lipid nanoparticles for enhanced bioavailability. As a prime example of an externally triggered, smart platform, AuNPs have emerged as particularly versatile and promising platforms for theranostic applications in ocular oncology [126].Their distinctive physicochemical properties including high surface-to-volume ratio, tunable optical behavior, and facile surface functionalization, allow simultaneous integration of diagnostic and therapeutic functionalities within a single nanoplatform [127]. Unlike viral vectors, which may trigger immune responses and insertional mutagenesis, AuNPs provide a biocompatible and customizable non-viral alternative for precise ocular gene delivery in malignancies such as uveal melanoma and retinoblastoma [128, 129].

AuNP-based theranostics are uniquely suited for complex ocular conditions involving concurrent cancer and glaucoma. Tumour growth in uveal or ciliary regions can obstruct aqueous humour outflow, elevating intraocular pressure (IOP) and leading to secondary glaucoma. In such dual-pathology scenarios, a multifunctional AuNP system could simultaneously deliver anticancer and glaucoma therapeutics. For example, an AuNP could be conjugated with anti-angiogenic agents to inhibit tumour vascularization while co-loading IOP-lowering drugs (e.g., Rho kinase inhibitors) or neuroprotective agents (e.g., neurotrophins). This unified platform offers targeted management of both malignancy and glaucomatous neurodegeneration.

Mechanistically, AuNPs facilitate enhancing drug and gene delivery through surface conjugation and targeted uptake. Their positively charged surfaces promote electrostatic interaction with negatively charged cell membranes, enhancing receptor-mediated endocytosis. Once internalized, environmental stimuli such as pH or enzymatic activity can trigger cargo release. Hybrid AuNP systems integrating DNA origami scaffolds with chemotherapeutic agents like doxorubicin have achieved superior intracellular accumulation and controlled release, resulting in improved cytotoxicity against ocular tumour cells [126]. Similarly, antibody- or aptamer-functionalized AuNPs enable receptor-specific targeting, for instance, binding to epithelial cell adhesion molecule (EpCAM) or folate receptors, thereby minimizing off-target effects and improving treatment specificity [130]. AuNPs have also been conjugated with siRNA, miRNA, and CRISPR-Cas9 complexes to silence oncogenic drivers such as BCL2 and GNAQ/GNA11, highlighting their promise for precision gene editing in ocular cancers. As mentioned above, the surface of AuNPs can be engineered with pH-sensitive polymers or thermo-responsive ligands, transforming them into smart drug delivery systems that release their cargo only upon encountering the acidic tumour microenvironment or in response to the localized heat they generate.

In diagnostics, AuNPs serve as potent contrast enhancers due to their strong surface plasmon resonance (SPR), which amplifies optical signals in imaging modalities like Surface-Enhanced Raman Scattering (SERS), computed tomography (CT), and fluorescence imaging [131]. For instance, Wang et al. (2023) demonstrated that AuNP-based SERS probes provided high-contrast imaging of uveal melanoma in preclinical models, enabling precise tumour delineation and real-time monitoring of treatment response.

Therapeutically, AuNPs can be activated by light for photothermal (PTT) and photodynamic therapy (PDT). In PTT, AuNPs absorb near-infrared (NIR) light and convert it into localized heat, selectively inducing tumour cell apoptosis while sparing healthy tissues [132]. A recent study using bipyramidal AuNPs (BiP-AuNPs) demonstrated that their sharp-tip geometry enhanced localized SPR, enabling efficient photothermal conversion and tumour ablation at lower laser power densities, minimizing trauma and preventing IOP elevation during ocular therapy [133].

In PDT, AuNPs conjugated with photosensitizers generate reactive oxygen species (ROS) upon light activation, causing oxidative damage and tumour cell death [134]. Functionalized AuNPs have been shown to enhance ROS generation and therapeutic efficacy in complex tumour models [135]. However, despite extensive data in colorectal and prostate cancers, no recent study has yet explored this actively targeted AuNP-PDT mechanism in ocular cancers such as retinoblastoma or uveal melanoma. This gap represents a critical opportunity for future research, as PDT could offer a spatially controlled treatment modality that minimizes collateral damage to the optic nerve, which is paramount in glaucoma-associated cases.

By integrating drug delivery, molecular imaging, and phototherapy, AuNP-based theranostics represent a paradigm shift for treating complex ocular diseases, particularly those associated with secondary glaucoma. Their tunable plasmonic properties, modular surface chemistry, and biocompatibility position AuNPs as an optimal platform for precision nanomedicine in ophthalmology. Future work should focus on optimizing AuNP parameters, size, charge, and coating to enhance ocular penetration, minimize inflammation, and improve safety. Combining AuNPs with biodegradable polymers and gene-editing tools may further advance personalized nanotherapies for vision preservation in ocular malignancies. Masse and co-authors have comprehensively reviewed the pharmacokinetics and pharmacodynamics of AuNPs in ocular drug delivery, offering valuable insights into their translational potential [136].

#### Potential of nanoparticle-based gene therapy

RNA interference (RNAi) has emerged as a potent therapeutic approach for ocular diseases by selectively silencing genes implicated in tumour progression or neurodegeneration [137]. Nevertheless, clinical application is limited by rapid degradation of naked RNA and restricted tissue penetration across the blood-retina barrier [138]. Nanoparticle (NP)-based systems have been designed to overcome these limitations by protecting RNA cargo and enabling targeted, stepwise delivery to the target cells.

Nanoparticle-mediated gene delivery operates through a coordinated series of events that ensure precise and efficient therapeutic action. Initially, nanoparticles accumulate at the target site via the enhanced permeability and retention (EPR) effect or through active targeting, where surface ligands such as antibodies or aptamers bind to disease-specific receptors (e.g., Epithelial cell adhesion molecule (EpCAM) or folate receptors). Following accumulation, cellular uptake occurs predominantly through endocytosis, facilitated by electrostatic interactions between the cationic nanoparticle surface and the negatively charged cell membrane. To prevent lysosomal degradation, advanced formulations incorporate cationic polymers such as polyethylenimine or chitosan that induce the “proton-sponge” effect, triggering endosomal swelling and rupture. This process enables endosomal escape and subsequent release of the RNA payload into the cytoplasm, where it engages the RNA-induced silencing complex to achieve gene silencing or modulation [139].

Gene therapy mechanisms in ocular oncology target key oncogenic pathways. Restoring tumour suppressors such as p53 can reactivate apoptosis, whereas silencing oncogenes such as GNAQ/GNA11 or anti-apoptotic genes (BCL2, Survivin) suppresses tumour proliferation. Additionally, anti-angiogenic RNAi therapies targeting vascular endothelial growth factor (VEGF) disrupt tumour vascularization, effectively starving the tumour of nutrients.

In cases of distal tumour-induced secondary glaucoma, NP-mediated gene therapy can simultaneously target both pathologies. A single nanoplatform may co-deliver siRNA against VEGF (to inhibit tumour angiogenesis) and siRNA or neuroprotective genes (e.g., BDNF) to prevent retinal ganglion cell apoptosis. Alternatively, silencing fibrotic genes in the trabecular meshwork could improve aqueous outflow, directly mitigating IOP elevation. Promising delivery systems include biodegradable PLGA nanoparticles and lipid-based carriers, which enable sustained siRNA release to posterior ocular tissues [140]. PLGA NPs encapsulating VEGF-siRNA have reduced angiogenesis in choroidal neovascularization models, while EGFR-targeted polymeric nanospheres promoted optic nerve regeneration in glaucoma models [141]. Advanced ternary siRNA complexes, combining siRNA, carriers, and tumour-targeting ligands, have achieved 50–60% tumour suppression by silencing GNAQ/GNA11 and BCL2 in uveal melanoma models [142].

Complementing these polymeric and lipid-based systems, AuNPs offer a unique convergence of gene delivery with real-time imaging and externally triggered therapy via localized surface plasmon resonance (LSPR). Their photothermal properties enable precise tumour ablation, while their surfaces can carry genetic or peptide payloads. For example, Kalmodi et al. used biosynthesized AuNPs conjugated with an HDM2-inhibiting peptide in Y79 retinoblastoma cells, leading to p53 stabilization and tumour suppression [143]. The efficacy of NP-mediated gene therapy can be enhanced by incorporating redox-responsive elements, such as disulfide bonds, into the polymer matrix to ensure rapid intracellular release of siRNA/CRISPR machinery upon exposure to the high glutathione concentrations in the cytoplasm.

The feasibility of RNAi-based nanotherapy has been clinically validated by the first FDA-approved siRNA drug for hereditary transthyretin amyloidosis, delivered via lipid nanoparticles [144]. Such milestones emphasize the immense potential of RNAi nanoplatforms in ophthalmology, paving the way for multi-functional ocular nanomedicine that integrates imaging, gene modulation, and neuroprotection to preserve vision.

#### Potential of virus-like particles

Developing efficient nanoscale systems for targeted delivery remains critical for overcoming ocular barriers and achieving precision therapy [145]. Vectors like non-viral and viral vectors facilitate the substances delivery, but they differ significantly in their properties. Non-viral NPs are categorized as inorganic, organic, or hybrid, with hybrid systems integrating different materials to enhance delivery efficacy. Their ability to evade immune responses and undergo surface modifications allows for targeted therapeutic applications. While engineered viral vectors (e.g., adeno-associated viruses, adenoviruses, and retroviruses) exhibit high transfection efficiency, their use is limited by pre-existing immunity and potential insertional mutagenesis [146]. In contrast, non-viral NPs provide safer alternatives due to their low immunogenicity, biocompatibility, and ability to co-deliver multiple therapeutic agents [147, 148].

Nonetheless, virus-like particles (VLPs) has been increasingly investigated in the recent years for ocular cancers, VLPs mimic the structural architecture of viruses but lack genetic material, eliminating any replication or infection risk [149]. This biomimicry enables efficient cellular entry and receptor targeting while avoiding the immunogenic drawbacks of live viral vectors.

The papillomavirus-derived VLP known as belzupacap sarotalocan (AU-011) exemplifies this innovation. AU-011 is conjugated with a photosensitizer that binds to heparan sulphate proteoglycans (HSPGs) on tumour cells [109]. Upon near-infrared light activation, it generates reactive oxygen species (ROS) that induce immunogenic cell death, exposing calreticulin and HSP90 to stimulate anti-tumour immunity [150]. Preclinical studies show high tumour selectivity and enhanced cytotoxicity in uveal melanoma cell lines, with BAP1-positive tumours demonstrating greater sensitivity [151].

In murine models, combining AU-011 with immune checkpoint inhibitors (ICIs) targeting PD-L1 and LAG-3 enhances tumour control at both primary and distant sites [150]. Clinical translation has been equally promising: the Phase 1b/2 trial (NCT03052127) achieved 55% tumour control and 91% visual acuity preservation, while subsequent AAO 2021 data reported 60% tumour control and 73% visual preservation after 12 months [152]. Building upon these results, the ongoing Phase II trial (NCT04417530) is evaluating suprachoroidal administration of AU-011 for primary indeterminate lesions and small choroidal melanomas, aiming to confirm its safety and efficacy as a minimally invasive, vision-preserving therapy [153]. In recognition of its potential, the U.S. FDA has granted AU-011 orphan drug and fast-track designations for ocular melanoma.

Importantly, the design principles of VLPs can be repurposed for glaucoma therapy. By re-functionalizing their surfaces with ligands targeting retinal ganglion cells (RGCs) or the trabecular meshwork, and replacing the photosensitizer with neuroprotective or gene-silencing agents (e.g., BDNF or anti-fibrotic siRNA), VLPs could simultaneously regulate IOP and prevent RGC loss.

Collectively, AU-011’s success highlights the transformative potential of VLP-based nanomedicine in ocular therapeutics. For a comprehensive review of the pharmacokinetic and pharmacodynamic principles that underpin VLPs platforms, readers are directed to Ma, S [154]. Future research should focus on biodegradable compositions and stimuli-responsive materials to enhance ocular retention, control release, and further improve safety. These developments firmly establish VLPs as a cornerstone of precision ophthalmology, bridging nanotechnology, gene therapy, and immunomodulation to protect and restore vision in patients facing the dual challenge of ocular cancer and glaucoma.

### Challenges in adopting multifunctional nanotherapeutics

By drawing on methodologies from cancer research, NP-based diagnosis and therapies for ocular cancer and associated conditions, such as glaucoma, have the potential for significant advancements. This versatile platform promises more targeted, less invasive, and potentially more effective treatment options. However, despite these promising developments, several challenges remain. Addressing these factors is critical to unlocking the full potential of these innovative approaches.

#### Targeting specificity in the ocular environment

Conventional chemotherapy faces significant challenges, including a lack of selectivity for tumour cells and multidrug resistance, which complicate drug delivery due to poor blood perfusion in tumour tissue [155]. Similarly, while radiation therapy is effective, it can cause both short- and long-term side effects that vary depending on the treatment area, other therapies, genetics, and lifestyle factors [156]. Achieving specific targeting of ocular melanoma cells without affecting surrounding healthy tissues remains difficult due to the complex and compact anatomy of the eye. For instance, the small size and delicate structure of ocular tissues limit the precision of drug delivery systems, while multiple protective barriers further complicate access to intraocular tumours [139].

Although NPs offer potential solutions for retinal drug delivery, their ability to penetrate retinal layers and maintain therapeutic concentrations remains limited due to factors such as the presence of tight junctions in the retinal pigment epithelium, the blood-retinal barrier (BRB), and rapid clearance mechanisms that hinder sustained drug retention [157]. The highly vascularized choroid can create inconsistent NP distribution due to its rapid blood flow, impacting delivery efficiency. Additionally, melanin in the uveal tract binds to NPs non-specifically, reducing their availability and compromising imaging contrast.

Targeting strategies must be carefully nano designed to recognize specific biomarkers on melanoma cells, such as integrins (e.g., α1β1 integrin) or glycoproteins (e.g., melanoma-associated chondroitin sulfate proteoglycan), while avoiding non-specific binding to melanin or other ocular tissues [158]. Failure to achieve precise targeting can prevent NPs from effectively reaching intraocular tumours, leading to off-target effects such as damage to healthy ocular tissues, immune system activation, or unintended accumulation in non-tumour regions. These effects compromise therapeutic outcomes by reducing efficacy and increasing the risk of adverse side effects [159]. Additionally, optimizing NP size, shape, and surface properties is essential to enhance permeability and retention within the ocular tumour microenvironment. For example, smaller NPs can navigate tighter ocular spaces, spherical NPs are less likely to undergo rapid clearance, and surface modifications such as PEGylation improve stability and reduce immunogenicity [160].

The inherent complexity and heterogeneity of tumours further limit the effectiveness of these systems. Variations in surface biomarker expression across different tumour regions can hinder uniform NP binding, while hypoxic zones in larger tumours may reduce the efficacy of drug-loaded NPs that rely on oxygen-dependent mechanisms.

#### Biocompatibility

Biocompatibility is critical for the successful application of NPs in drug delivery and ocular imaging. To be clinically viable, NPs must avoid inducing toxicity or immune reactions. For instance, the shape and curvature of NPs significantly influence their circulation and toxicity; gold nanorods are more hazardous than spherical particles due to these physicochemical differences [161]. Poorly engineered NPs may provoke immune responses, inflammation, or unintended accumulation, which is particularly problematic in cancer immunotherapies, where biocompatibility is essential to prevent complications such as cytokine storms or autoimmune reactions [162].

The delicate physiology and unique protective barriers of the eye further emphasize the importance of biocompatibility. While the nanoscale size of NPs allows them to bypass these barriers non-invasively and deliver therapeutic agents to the posterior segment of the eye, this advantage comes with risks such as ocular toxicity, inflammation, and retinal damage. Some studies indicate that gold NPs do not harm retinal integrity, while others report toxicity. Similarly, silver NPs, known for their antimicrobial properties, can cause DNA damage and arrest cell cycles in retinal cells [163].

Polymer-based nanomaterials, such as chitosan, exemplify the dual challenges of adaptability and biocompatibility. Although chitosan is a promising candidate, it suffers from poor hemocompatibility and off-target distribution. For example, chemotherapeutic-loaded chitosan NPs are easily phagocytosed by the reticuloendothelial system (RES), leading to unintended organ delivery [164, 165]. Inorganic NPs, such as silver and zinc oxide, also exhibit variable toxicity depending on size, shape, and functionalization. Silver NPs induce oxidative stress and apoptosis in corneal cells, while zinc oxide NPs cause neurotoxicity and oxidative stress [166–170].

Despite advances, many nanodrugs reduce chemotherapy toxicity without significantly improving therapeutic outcomes. Testing NPs in models that simulate chronic diseases could better reflect real-world conditions. To ensure safety and efficacy, rigorous preclinical testing, careful material selection, surface modifications, and precise dosage optimization are necessary to mitigate adverse effects and achieve therapeutic goals in ocular imaging and therapy.

#### Immune response

The immune-privileged nature of the eye provides some protection against inflammation and immune responses, but it does not entirely shield against the challenges posed by NPs. Immune cells, such as microglia and macrophages, can respond to NPs, triggering inflammation within the ocular environment. Additionally, NPs can be cleared through aqueous humour outflow or phagocytosis by resident ocular cells, reducing their retention time and therapeutic effectiveness. Therefore, designing NPs that minimize immune activation while maximizing retention within the tumour microenvironment is crucial for improving imaging and therapeutic efficacy.

NPs have demonstrated their potential to enhance anticancer drug delivery selectively [171]. Leveraging the enhanced permeability and retention (EPR) effect, particles smaller than 200 nm can accumulate in tumours due to leaky vasculature and poor lymphatic drainage. Controlled drug release mechanisms, triggered by factors such as ultrasound, pH changes, heat, or particle composition, further augment therapeutic potential. However, immune responses may clear NPs from circulation or redirect them to other organs, such as the liver, spleen, or BRB. The tight junctions and high metabolic demands of the BRB pose additional obstacles, often preventing effective NP delivery to intraocular tumours and increasing the risk of immune rejection [172].

Immune clearance further challenges NP-based drug delivery by reducing drug encapsulation efficiency and causing unintended cytotoxic drug release [145, 173]. These effects diminish therapeutic efficacy and increase the risk of adverse side effects. Addressing immune clearance and designing NPs with properties that enhance ocular retention while minimizing systemic immune reactions are critical for successful drug delivery.

#### Retention and clearance mechanisms

Ocular drug delivery is hindered by rapid clearance mechanisms. Tear turnover continuously flushes away foreign particles, while immune cells actively eliminate NPs. The tear film, composed of water, mucins, and oils, acts as a protective barrier, further expelling foreign matter [160]. Additionally, the cornea’s multi-layered structure and the blinking reflex contribute to NP removal [174].

While prolonged NP retention may enhance therapeutic efficacy, it also carries risks of toxicity and inflammation. Smaller NPs may escape through drainage pathways, whereas larger or more rigid particles can persist, potentially causing adverse effects [175]. Achieving a balance between retention and clearance is essential for successful ocular drug delivery.

Biodegradable NPs, such as those composed of PLGA or chitosan, offer promising solutions. These materials degrade into non-toxic byproducts that can be naturally eliminated through drainage pathways [176]. For instance, PLGA NPs have been successfully used to deliver corticosteroids for uveitis, enabling sustained drug release without prolonged accumulation.

#### Translational challenges

The global nanomedicine market is projected to experience substantial growth in the coming years. Valued at approximately $171.7 billion in 2020, it is expected to reach $393 billion by 2030, with a compound annual growth rate (CAGR) of 9.2% from 2021 to 2030, according to Allied Market Research [177]. This growth is driven by the rising prevalence of chronic diseases, advancements in nanotechnology, and an aging global population [178]. Additionally, the development of innovative drug delivery technologies, the diverse applications of nanomedicine in healthcare, and the increasing demand for safe, cost-effective therapies further contribute to market expansion. However, barriers such as lengthy approval processes and environmental risks associated with nanomedicines could hinder progress.

Numerous studies have explored nanotherapeutics for ocular diseases, but their complexity compared to traditional treatments has limited commercialization. These complexities lead to lengthy and costly regulatory approval processes [83]. The aggressive and rare nature of cancers such as uveal melanoma further complicates clinical trial design due to small patient populations and the limited availability of animal models that accurately replicate human ocular physiology [179]. Common preclinical models, such as rodents and rabbits, while cost-effective, exhibit significant anatomical and physiological differences compared to humans [180]. For example, variations in eye size, blinking rates, and immunological features affect drug pharmacokinetics, reducing the predictability of these models for human outcomes.

Scaling up NP manufacturing presents additional challenges. Maintaining batch-to-batch consistency is difficult due to variations in particle properties that influence therapeutic efficacy. High-energy techniques such as ultrasonication and hot homogenization can improve stability but may alter thermodynamic properties and increase production costs, further hindering scalability [181]. Ensuring both quality and affordability in NP production is critical for clinical adoption.

Regulatory hurdles also arise because many NP systems, such as multimodal imaging and theranostic NPs, do not fit neatly into existing guidelines. For example, the U.S. FDA may require a new submission process for combination products, while the EMA may classify them under both drug and medical device regulations, adding complexity to the approval process [182]. This dual regulatory requirement can lead to delays and increased costs for developers. As a result, these systems require tailored regulatory pathways that address both diagnostic and therapeutic standards, which are often costly and time-consuming.

## Conclusion and future perspectives

The complex interplay between glaucoma and ocular cancers poses significant diagnostic and therapeutic challenges, often rendering conventional treatments inadequate due to their lack of specificity, potential for off-target toxicity, and inability to address both conditions simultaneously. This review has highlighted the transformative potential of nanotherapeutics in this domain. Key findings demonstrate that engineered nanoparticles can overcome fundamental ocular barriers, enabling targeted drug delivery, enhanced imaging for early detection, and the integration of diagnostic and therapeutic functions into unified theranostic platforms. From AuNPs facilitating photothermal ablation to lipid-based carriers enabling gene therapy and virus-like particles offering precise tumour targeting, these innovations provide a compelling strategy for concurrent tumour suppression and glaucomatous neuroprotection. By directly targeting the shared pathological mechanisms of these diseases, nanomedicine offers a promising pathway toward integrated management strategies that could profoundly improve vision preservation and patient survival.

Looking ahead, the successful translation of these nanotechnological advances from the laboratory to the clinic hinges on a concerted, interdisciplinary focus on several critical fronts. Firstly, future research must prioritize the intelligent design of nanoparticles to optimize their ability to navigate the eye’s intricate biological barriers such as the blood-retinal barrier and evade rapid clearance mechanisms. This involves refining properties like size, surface charge, and the use of bioactive ligands for targeted uptake. Secondly, the development of advanced multifunctional platforms that combine real-time, high-resolution imaging with controlled, multi-drug delivery is essential for creating personalized and adaptive treatment regimens. A parallel and equally critical endeavour is to establish a robust safety framework through rigorous, long-term toxicological studies of both organic and inorganic nanomaterials, with a strong emphasis on developing biodegradable carriers to mitigate chronic toxicity concerns. Bridging the translational gap will also require the development of more predictive disease models and scalable, GMP-compliant manufacturing processes to ensure batch-to-batch consistency and clinical viability. Finally, the convergence of nanomedicine with cutting-edge fields like gene editing (e.g., CRISPR-Cas9) and AI-driven design paves the way for truly personalized therapies, where nanoparticles can deliver gene-based treatments to correct oncogenic mutations while providing neuroprotective support [183]. By systematically addressing these research directions, the field can unlock the full potential of nanotherapeutics, ultimately redefining the standard of care for patients with vision-threatening, glaucoma-associated ocular malignancies.

## Data Availability

No datasets were generated or analysed during the current study.
